# Anatomy and Physiology

**Published:** 1858-01

**Authors:** 


### ANATOMY AND PHYSIOLOGY.

1. NEW VIEWS ON THE PHYSIOLOGY OF THE LARGE INTESTINE. By *M. F. Colby, M.A., M.D., etc.*, Stanstead, C. E.—IT is now more than eighteen months since I discovered the error in the received physiology of the function of the large intestine, particularly in that part of it called descending colon, sigmoid flexure, and rectum. Every day's observation since has confirmed me in the correctness of my views. Although I have not been able to engage in general practice, I have had numerous opportunities of testing them as to their bearing on pathology. The knowledge of the true function of the descending bowel does away with all the uncertainty complained of by medical men as to the effect of cathartics, and more particularly of enemas, in many cases. A discussion took place in the Westminster Medical Society, in 1833, which is reported in the *London Lancet.* The discussion developed one fact, that there was a consciousness among all present that there was something not satisfactory in the received physiology; which led to the question by the anatomists present, whether there was anything in the anatomical structure of the descending bowel which could operate as a valve?

I can demonstrate the received physiology of the function of the descending bowel to be untenable, and that it implies the charge that the Creator has left a defect in the organization of a particular part, which renders it inadequate to the performance of the function assigned it. My new physiological doctrine recognizes two distinct apparatuses, each possessing peculiar and distinct functions over and above what is recognized by the old system. These functions were supposed to pertain to that apparatus called the large intestine, and heretofore assigned to the function of organic life, assisted by the voluntary cooperation of the abdominal muscles.

As to the purport of my new physiological doctrine, I quote from lectures which I am preparing, illustrative of the subject, the following recapitulation:—

1st. I assume that the organic function of the colon ceases at its left transverse extremity.

2d. That the portion called descending colon and sigmoid flexure has a separate and independent function.

3d. That this portion of the bowel is anatomically inadequate to the performance of the function heretofore assigned it.

4th. That this portion constitutes the link between the animal and organic life. That it is possessed of both animal sensibility and contractility to such an extent as to entitle its functions to be considered those of animal life.

5th. That although it is to a certain extent subject to the will, and can be brought into action at any moment by it, yet it has an independent instinctive life, which gives it an influence and a power which neither its organic or its animal life could give it.

6th. I assume the name of curator as proper to express its function; and as it is a dualite acting under its instinctive life, at times in a separate capacity, I give the name curator superior to that portion above the superior spinous process of the ilium, and which for the time is devoted to the functions of organic life; and curator inferior to the portion below, usually called sigmoid flexure—this for the time being devoted to the functions of animal life.

7th. That the curator, when acting as a unit, occupies the post of observation between the two lives. That it takes cognizance of the time when the digestion and the nutritive absorption is completed in the small intestine; that it then opens the ileo-colic valve, and at the same time, by a suctive and expansive action, it takes the fæcal matter from the transverse colon and conveys it to the rectum, which it aids the levator ani muscles to raise, and by a divergent action of its two longitudinal muscles it opens to receive the same. The curator, by its instinctive power, recognizes the fitness of the rectum to receive and expel the fæcal matter simultaneous to the opening of the ileo-colic valve; it also at the same time brings into action the abdominal muscles, by which the contents of the small intestine are pressed forward to supply the place of the refuse matter removed from the colon. Its office is therefore not only prehensile in taking the fæcal matter from the transverse colon and conveying it to the rectum, but it exercises the conservative function of keeping the ileo-colic valve closed till such time as the absorption of all nutritive matter from the contents of the small intestine renders its closure no longer necessary.

8th. That the rectum is part of an apparatus which I call rectal, and which is wholly under the domain of the will; that it exercises the function of defæcation, and aids in that of urination and parturition. In its anatomical structure it is analogous to that of the upper part of the digestive tube, with the difference of the reversion of the sphincters. It consists of the strongest muscular portion of the bowel; the rectum, with its muscles; the two sphincters, the levator ani, the coccygei, etc. The same looseness of the cellular tissue, which connects the mucous with the muscular coat of the œsophagus, is found between these coats of the rectum.

9th. That the power of the will extends over that part of the digestive tube which extends from the mouth to within two or three inches of the cardiac orifice of the stomach;. so also the power of the will extends from the external sphincter ani to within two or three inches of the left transverse extremity of the colon.

10th. That the rectum, in that abnormal state which results from phlogosis of its muscular coat, has its contractility exalted so as to cause it to act antagonistically to the curator. This is the most frequent cause of constipation and its consequences. When this contractility becomes spasmodic, this resistance leaves the curator to mechanical forces,—hence result accumulations and distension of its weakened side-walls. It is this abnormal state of the most sensitive part of the digestive tube which fills the hospitals with the insane. It is also in this state that the curator, by its instinctive life, acts as a dualite by a peculiar transposition which gives it a great power in overcoming the resistance of the rectum.

11th. The ileo-colic valve may have its function suspended by local disease as well as by peritoneal inflammation; but the most frequent cause is the suspension of the function of the curator, which may arise from antagonism from the abnormal state of the rectum, or from a phlogosed state of its own mucous membrane. A sudden closure of the valve would cause tympanites, ileus or strangulated hernia. A weakened or too active state of the valve would result in emaciation from the premature passing of the nutritive matter.—*Montreal Medical Chronicle*, Aug. 1857.

2. RESEARCHES ON THE HISTOLOGY OF THE NERVOUS SYSTEM. By *M. Jacubowitsch.*—Professor Jacubowitsch, a young Russian *savant*, has been, during the last four years, laboriously employed in the microscopical study of the nervous centres. An account of these has been recently presented by M. Flourens with almost extravagant eulogiums to the *Académie des Sciences*, before which learned body the author also exhibited several beautiful drawings from some of his preparations, said to amount to 25,000 in number. As M. Jacubowitsch is about shortly to publish a full account of his researches, and is also, we believe, soon to visit London in order to exhibit his preparations, we shall at present content ourselves with the following *resumé* of his communication to the Académie, drawn up by himself and by M. de Castelnau.

1. The cerebro-spinal system is composed of three descriptions of cells, which, while assuming the essential form of the animal cell in general, are distinguished by the following special characteristics:—2. Axis-cylinders arise from the periphery of these cells, which they distinguish from each other by their number. (*a*) In the first species, or multipolar cells, the number of axis-cylinders radiating from the periphery varies from 3 to 8, and is habitually 3 or 4. The form of this cell is irregular (*b*) in the second species, or fusiform cells; the axis-cylinders are 3 in number, sometimes 4, but never more. They assume the spindle form, and are much smaller than the multipolar cells. (*c*) In the third species, or bipolar cells, the axis-cylinders are but two in number. These cells are of an oval form, and in size are intermediate between the two other species. 3. (*b*) The multipolar cells are met with in the medulla spinalis, in the cerebellum, and in the corpora quadrigemina. It is remarkable that none are found in the medulla oblongata. In the medulla spinalis they are situated in the anterior cornua and in the gray substance. (*b*) The fusiform cells are found in the medulla spinalis and oblongata, in the cerebellum, in the corpora quadrigemina, and in all parts of the cerebral hemispheres, which are almost entirely formed of them. In the medulla spinalis these cells are placed in the posterior cornua. (*c*) The bipolar cells are found in the medulla spinalis and oblongata, in the cerebellum, and in the corpora quadrigemina; but the cerebrum does not contain any. In the medulla spinalis they are principally found around the central canal. They are very abundant in the medulla oblongata, constituting its greatest portion. 4. (*a*) The axis-cylinders proceeding from the multipolar cells form the nerves of motion; and it is for this reason that the author terms these cells cells of motion. (*b*) The axis-cylinders of the fusiform cells form the nerves of sensation, and the cells are termed cells of sensation. (*c*) The axis-cylinders of the bipolar cells are in relation with the grand sympathetic; and the cells are termed ganglionary cells. 5. The same and different species of cells communicate with each other as follows:—(*a*) The cells of motion communicate from one side to the other by the anterior commissure, or rather this commissure is formed by the intercrossing in every direction of the axis-cylinders which proceed from these cells. Those of the same side also communicate by the axis-cylinders, which proceed directly from one to the other. (*b*) The cells of sensation communicate from one side to the other by the posterior commissure, or rather they constitute that commissure; but they remain parallel to each other, and do not intercross. Those of the same side communicate directly. (*c*) The axis-cylinders of the ganglionary cells pass by either the anterior or the posterior commissure; and thus communicate from one side to the other, like both the other species. Communications also take place between those of the same side. (*d*) Communications, moreover, take place between the different varieties of cells; but the author has only met with them in the cerebellum, and in the corpora quadrigemina, in which the three varieties of cells occur. 6. The different species of cells are not equally abundant in all the regions of the medulla spinalis. Thus, in the cervical and lumbar regions the cells of motion predominate, while in the dorsal region those of sensation are most numerous. 7. The medulla oblongata is purely and simply a prolongation of the medulla spinalis, a prolongation entirely formed of cells of sensation, containing in their centre a nucleus of ganglionary cells. 8. The cerebellum is formed of four elements,—viz. (*a*) of three elements, constituting the white substance, and which are prolongations by means of the peduncles of the pyramidal bodies which furnish the elements of the cells of motion; of the restiform bodies, which furnish the elements of sensation; and of the ganglionary element, which enters with each of the other two. (*b*) Besides these three elements, the periphery, or cortical substance of the cerebellum, is formed of characteristic pyriform cells peculiar to this organ. From these pyriform cells prolongations are sent to the surface of the organ, forming a layer termed by the author the *couche en baguettes*. 9. The corpora quadrigemina, formed by the prolongations or the peduncles of the cerebellum, by the restiform bodies, and by the horse-shoe shaped commissure, contain the three varieties of cells which they derive from these various origins. But this is the last point where these three varieties are found together. Above this, in the hemispheres, as already stated, the cells of sensation, of which the corpus callosum forms the commissure, are alone met with. 10. With the exception of the optic, the olfactory, the auditory, the vagus, the glosso-pharyngeal, and the hypoglossal nerves, which only consist of cells of sensation and ganglionary cells, all the nerves are formed of the three varieties of cells, with this difference among them, that the nerves of motion contain especially the cells of motion, and so on. 11. Besides the elements now described, the cerebro-spinal system contains a special connective tissue uniting the other elements, and which differs from the connective tissue of other organs by not containing any cells. It is, moreover, in infinitely small proportion as compared with the nervous tissue, and seems to play no other part than that of a bond of union. 12. These three categories of cells vary in different species of animals. They are smallest in size in man, and in general they are small in a proportionate degree to the intellectual development. The number of the nervous cells bears no proportion to the size of the brain.

From this brief *resumé*, M. de Castelnau observes, we are enabled to deduce some of the principal consequences of the fact upon which M. Jacubowitsch's researches are based.

1. The three entirely distinct functions of the nervous system—motility, sensibility, and organic action—are not only exercised by three different orders of nerves, but, moreover, these orders of nerves take their origin in different central anatomical elements.

2. The medulla spinalis, whence spring the general nerves, contains equally and naturally the three orders of elements: but the brain, the organ of the intellect, contains but the elements of sensibility, and the nerves of the special senses are also made up of the same element, with which is united in small proportion only the ganglionary element.

3. The element of sensibility is of different dimensions in man and animals, being smaller in the former and larger in the latter; so that for even an equal cerebral mass the human brain is much more rich in sensible elements than is that of animals. From this to the relative richness of each particular man, and especially of each human race, the distance does not seem insurmountable. Will it ever be passed? That is a question we must reserve, but still with a doubt accompanied by much hope.

4. M. Jacubowitsch's researches scarcely promise less to the pathologist than to the physiologist. They have already shown that in affections of the nervous system, in which the most minute ordinary examination could detect no material lesion, such lesion was nevertheless considerable, since one or more of these orders of elements had become greatly altered in their form, or even had undergone destruction. This single result sufficiently indicates the brilliant horizon that may be opening to the pathological anatomist. If it exhibits to us clearly but a small part of that which we now seem to have a glimpse of, M. Jacubowitsch will not have rendered less service to the practitioner than to the physiologist and the true philosopher.—*Moniteur des Hôpitaux*, No. 108, and *Comptes Rendus*, tome xlv. No. 9.—*Med. Times and Gazette*, Oct. 17, 1857.

3. EFFECT OF RED BLOOD ON THE VITAL PROPERTIES OF THE CONTRACTILE AND NERVOUS TISSUES.—Two years ago, Dr. Brown-Séquard published the results of numerous experiments, showing that red blood—*i.e.* richly oxygenated blood—has the power of reproducing the vital properties of almost all the contractile tissues, when it is injected in the artery some time after these tissues have lost these properties. In a paper read to the Academy of Sciences on the 19th of October last, this physiologist relates some new facts on this subject. It is well known, since the admirable experiments of Sir Astley Cooper, that animals die from asphyxia when circulation is stopped in their four encephalic arteries, and that if the circulation be quickly re-established, the animals recover almost immediately. Dr. Brown-Séquard has ascertained that if circulation takes place again a few minutes after the last respiratory movement, life does not reappear; but if the lungs are insufflated, the apparently dead trunk revives, and becomes endowed with a very energetic reflex power; and that five, ten, or fifteen, and in one case seventeen minutes after the last respiration, in insufflated dogs, if circulation is re-established in the encephalon, the functions of the brain proper and of the respiratory nervous centre reappear, and the animal may be restored to full life. In heads separated from the body, injections of richly oxygenated blood may reproduce the actions of the brain proper and of the medulla oblongata many minutes (even fourteen or fifteen) after decapitation.

In a second part of his last paper, Dr. Brown-Séquard points out a radical difference between the two kinds of blood, the arterial and the venous—or rather, the red and the black. He has ascertained by a great many experiments that red blood—*i.e.* blood charged with oxygen, whether arterial or venous—never has the power of stimulating or exciting any organ or tissue; while black blood—*i.e.* blood charged with carbonic acid—has the power of stimulation in a very high degree as regards the nervous centres, and in a lower degree as regards the sensitive and motor nerves, and the contractile tissues. On the contrary, the red blood has the power of regenerating the vital properties, while black blood is hardly able to maintain them at a low degree. Dr. Brown-Séquard calls attention to a peculiar mode of action of black blood, which consists in its producing intermittent or periodical effects, and sometimes perfectly rhythmical actions, even in the muscles of animal life. He finishes his paper by the indication of the danger of employing black blood in transfusion. He relates facts to prove that the blood of an animal acts as a poison when injected in the veins of an animal of another species, only when it is black and charged with carbonic acid. The blood of a rabbit may kill the same individual as well as another animal of the same species, if it is injected black—whether defibrinated or not—in its veins; so it is for dogs, for cats, for birds; while, on the contrary, richly oxygenated blood—whether venous or arterial, and defibrinated or not, and taken from birds, turtles, etc.—may be, without any ill effect, injected in the veins of a mammal. The great danger of transfusion of blood, after the entrance of air in the veins and the coagulation of its fibrin, is, therefore, the employment of a liquid containing too much carbonic acid. This danger, and at the same time the danger of coagulation, may easily be avoided by employing whipped venous blood, which, during the operation of whipping, loses its coagulating principle and much of its carbonic acid, and absorbs a good deal of oxygen.—*Med. Times and Gazette*, Nov. 7, 1857.

4. On the Structure of the Nervous Centres. By *Professor Lenhossek.* The following is a literal translation of an abstract of the paper furnished to the Académie des Sciences by M. Lenhossek, giving an account of his collection of anatomical preparations, made after the method of Mr. Lockhart Clarke, F.R.S., and with which, we understand, he has just arrived in London:

"It results from my researches—1. That the central nervous system is composed of gray substance, of white substance, and of an intermediate substance termed the gelatinous substance of Rolando. 2. That the gray substance is formed of a general hyaline mass, having three kinds of nerve-cells—(*a*) Nerve-cells with all their attributes, generally diffused; (*b*) Large Nerve-cells with all their attributes, united into groups. These are seen in the motor columns, in the sensitive columns, and elsewhere. (*c*) Capherical nerve-cells with all their attributes, filled with a deep-brown pigment. These form only the ferruginous substance and the black substance of Soemmering. 3. That the white substance is formed of primary fibres, which terminate in the form of radiations in the different organs of the central nervous system. 4. That the so-called gelatinous substance is formed by the gray substance, which traverses, in the form of network, the bundles of the white substance. 5. That the gray substance has the following relations:—(*a*) In the spinal marrow it constitutes four columns, two anterior motor and two posterior sensitive, united together by the gray commissure. (*b*) In the medulla oblongata these four columns change their relative position, the anterior columns becoming internal, and the posterior external. They preserve this juxtaposition throughout the extent of the rhomboidal sinus. Farther on, the motor columns are continued alone to the bottom of the third ventricle, terminating in the infundibulum. The sensitive columns pass into the optic thalami and the corpora striata. (*c*) At the point where the juxtaposition of the four columns takes place, there the commissure disappears, and the median cloison of Vicq d'Azyr commences, this being formed by a prolongation of the gray substance. This cloison is continued through the entire length of the pons varolii. 6. That in the spinal marrow the white substance of the one side is completely separated from that of the opposite side by the anterior and posterior longitudinal groove; and in the medulla oblongata and pons varolii an analogous separation is produced by the cloison of Vicq d'Azyr. 7. That at the point where the juxtaposition of the four columns of the gray substance takes place, the white substance proceeds more and more forward until the gray substance at last remains exposed in the rhomboidal sinus. 8. That the primary fibres of the roots of all the nerves arise in the gray substance. (*a*) In some cases these fibres proceed from prolongations of the nerve-cells. (*b*) But most commonly they arise in groups, without its being possible to determine their origin. These fibrous groups traverse the white substance in different directions, in order to form the roots of the nerves at the surface. They never contribute to the development of the white substance, nor does the latter furnish any fibres for the roots of the spinal nerves. 9. That the motor roots of the spinal nerves and of the motor cerebral nerves, such as the hypoglossal, the external and internal motors of the eye, the facial, the small portion of the trigeminus, and the common ocular motor, take their origin only in the motor columns. 10. That the sensitive roots of the spinal nerves and of the sensitive cerebral nerves—as the auditory, the large portion of the trigeminus, the optic, and olfactory—proceed only from the sensitive columns. 11. That the roots of the mixed cerebral nerves—as the two superior roots of the accessory nerves of Willis and the pneumo-gastric—arise in both the motor and sensitive columns. 12. That there are four kinds of crossings in the spinal marrow—medulla oblongata and pons varolii. (*a*) In the spinal marrow the primary fibres of the motor roots cross in front of the central canal, and the fibres of the sensitive roots cross behind it. These intra-crossings are produced by a portion of the primary fibres of the roots taking their origin in the opposite side. (*b*) In the medulla oblongata and pons varolii, the primary fibres of the roots of the cerebral motor nerves, and only the motor portion of the mixed cerebral nerves, intercross at the middle of the motor columns, for the same reason as in the spinal marrow. (*c*) In the cloison of Vicq d'Azyr there is an intercrossing from right to left of some of the fibres of the white substance of the medulla oblongata and of the pons varolii. (*d*) There is a crossing of the six walls of the bundles of the white substance and of the medulla oblongata, in front of the central canal, known under the name of the pyramidal decussation. 13. That the primary fibres of the roots of the nervous plexuses of the pia mater, as also those of all the roots of the accessory nerves of Willis, except the two superior roots, proceed from all the periphery of the gray substance. In the plexuses at the surface of the pia mater are found—(*a*) Nerve-cells interposed between the primary nervous fibres. (*b*) Nerve-cells in groups, suspended and floating at the external surface of the nerves of the pia mater: these latter are filled with pigment. 14. That the two olivary bodies are composed of two substances—an external gray, having convolutions, and an internal white. The white substance is formed by the irradiation of the primary fibres of the peduncles of these bodies, which take their origin in the motor columns, and by the transverse commissure which traverses the cloison of Vicq d'Azyr. 15. That the central canal of the spinal marrow runs along its entire length, and opens into the calamus scriptorius. The walls are formed internally by a covering of cylindrical epithelial cells, and externally by a layer of the longitudinal fibres of Clarke, which extend into the epithelial layer of the rhomboidal sinus. In the lumbar region a granular mass is interposed between this fibrillary layer and the epithelial cells. 16. That on each side of the central canal there is a large vein, which successively bifurcates in the region of the medulla oblongata and in that of the medullary cone."—*Moniteur des Hôp.*, No. 129. *Ibid.*

